# The attitude and acceptability towards medical promotional tools and their influence on physicians’ prescribing practices in Jordan and Iraq: a cross-sectional study

**DOI:** 10.1186/s12913-022-07525-1

**Published:** 2022-01-25

**Authors:** Karrar Ehsan Ali, Abdallah Y. Naser, Rabaa Al-Rousan, Hassan Alwafi, Amal Khaleel AbuAlhommos, Zahra Khalil Alsairafi, Emad M. Salawati, Mohammed Samannodi, Mohammad S. Dairi

**Affiliations:** 1 Department of Applied Pharmaceutical Sciences and Clinical Pharmacy, Faculty of Pharmacy, Isra University, Amman, Jordan; 2 Inpatient pharmacy department, General Hospital in Al Diwaniyah, Al Qadisiyah, Al Diwaniyah, Iraq; 3 Faculty of Medicine, Umm Al-Qura university, Mecca, Saudi Arabia; 4 Pharmacy Practice Department, Clinical Pharmacy College, King Faisal University, Alhasa, Al Hofuf, Saudi Arabia; 5 Department of Pharmacy Practice, Kuwait University, Kuwait, Kuwait; 6 Family medicine Department, Faculty of medicine, King Abdulaziz university, Jeddah, Saudi Arabia

**Keywords:** Iraq, Jordan, Medical representative, Pharmaceutical, Practice, Prescribing, Promotion

## Abstract

**Background:**

Pharmaceutical companies spend more than one-third of their sales revenue on marketing and promotion directed toward healthcare professionals. There has been a focus on the relationship between healthcare professionals and the pharmaceutical industry in recent years. This study aims to explore the attitude toward and acceptability of medical promotional tools and their influence on physicians’ prescribing practices in Jordan and Iraq.

**Methods:**

A cross-sectional survey study was conducted to explore the influence of visits by medical representatives (MRs) and medical promotions on physicians’ prescribing practices between June and October 2020 in Jordan and Iraq. Previously validated questionnaires were used.

**Results:**

A total of 801 physicians completed the questionnaires. Face-to-face visits, followed by the dispensing of medical samples, were the two most common promotional methods used by MRs. 48% of participating physicians reported that they would accept the promotional marketing tools offered to them. MRs focused on the key selling points of their product during medical promotions, and 39.6% of the physicians reported that MRs had a negative attitude toward their competitors’ products. 69.9% of the physicians reported that they would change their practice after participating in conferences or meetings.

**Conclusion:**

Medical promotional tools have a clear influence on physicians’ prescribing practices in Jordan and Iraq. Therefore, medical promotion should be controlled and guided by clear and country-specific ethical guidelines. This will ensure safe medical promotion to physicians and optimise the healthcare practices provided to patients.

**Supplementary Information:**

The online version contains supplementary material available at 10.1186/s12913-022-07525-1.

## Background

The use of medications contributes greatly to improving and maintaining people’s health, including curing acute illness, relieving signs and symptoms, managing chronic disease and preventing future diseases [1]. However, deciding which drug to use for the illness involves weighing potential benefits over possible harm. To make an informed decision as to which drug should be used, there is a need for information about the goals of the therapy, the way to use it properly, how it works, the benefits and adverse effects, how this medicine compares to other available treatment and also the relative cost-effectiveness [2, 3].

Two main sectors serve the healthcare system in Jordan: the public and the private sector. A significant portion of the Jordanian population also receives healthcare through programmes led by the United Nations and non-governmental humanitarian agencies.

Primary healthcare centres in Jordan provide various types of quick medical services, including vaccination, chronic disease management services, and maternity and child care [4]. Around 70% of the Jordanian population and 55% of the Kingdom’s overall population are covered by health insurance. In 2012, the total healthcare expenditure reached 7.58% of the gross domestic product (GDP), with over 60% of the spending in the public sector [4].

The Iraqi healthcare system is mainly central, with a specific proportion of government funding directed toward this sector yearly. According to the World Bank, the Iraqi government spending on healthcare increased from 2.7% of the GDP in 2003 to 8.4% in 2010 [5, 6]. Despite this, only 3.6% of the Iraqi population are covered by health insurance and currently there is no health insurance system to serve the general public [5].

Physicians, pharmacists, and other healthcare professionals play a key role in prescribing appropriate medications and ensuring that they are used properly [7]. Due to growing concerns over medication safety, there has been a focus on the relationship between healthcare professionals and the pharmaceutical industry in recent years, particularly the influence of the pharmaceutical industry on prescribing decisions due to various promotional methods that may affect prescribing patterns [8–10]. This effect can lead to less than optimal medication choices, which may sometimes adversely affect patient health [11].

Pharmaceutical companies spend more than one-third of their sales revenue on marketing and promotion to retain and maximise their market share [12]. Companies use several effective methods to promote their products, mainly through formularies and medical journals and presentation of new drugs at conferences and workshops. Other promotional tools used include sending medical representatives (MRs), online and direct mail contact with physicians, giving free drug samples to distribute to patients, and low-cost or high-cost gifts [12, 13].

There is evidence that during related pharmaceutical advertising campaigns the rates of diagnosis of specific conditions increase, but the question remains to what extent these conditions require medical treatment and whether the increase in diagnoses is due to benefits in the form of incentives provided by pharmaceutical companies [14, 15]. If the threshold for diagnosis of a health condition shifts to include minor health problems, increased rates of diagnosis and treatment do not necessarily lead to health benefits [2]. A previous study in Jordan reported that the acceptance of pharmaceutical companies’ gifts by physicians is at a high level, and statistically there is a substantial impact of pharmaceutical companies’ gifts on physicians’ prescribing behaviour and practices [16]. Another study in Lebanon confirmed the fact that interactions between physicians and MRs are frequent. While these interactions may be beneficial, they can also negatively impact drug prescription and dispensing practices [17]. In Iraq, a study found that Iraqi physicians accept various types of gifts, free samples and sponsored conference invitations from pharmaceutical companies, and this can impact physicians’ prescribing practices and result in early acceptance of new medications based on promotional information, even in the absence of clinical evidence about the drug’s safety and efficacy. This unethical interaction between physicians and pharmaceutical companies may have a negative effect on physicians’ prescribing practices and, in turn, may result in unwanted consequences for the patients and may negatively affect patients’ health [18].

The aims of this study is to explore the attitude toward and acceptability of medical promotional tools and their influence on physicians’ prescribing practices. Additionally, this study will explore the characteristics of medical promotion provided by pharmaceutical companies through their MRs to physicians in Jordan and Iraq.

## Methods

### Study design

A cross-sectional survey using a self-administered questionnaire was conducted for the period between June and October 2020 in Jordan and Iraq.

### Sampling strategy

Practising physicians in public and private health facilities who were willing to participate in the study formed the study population. A convenience sampling technique was used to recruit eligible participants. This is a non-probability sampling technique in which participants (physicians) from the target population who meet the inclusion criteria of the study and who are easily accessible due to availability at a given time, geographical proximity, or willingness to participate in the study are included.

### Participants’ recruitment

Practising physicians were approached and requested to participate in the study. In order to increase the number of participants in this study, two recruitment procedures were applied. The questionnaire tool was distributed face-to-face by visiting physicians at their practice site (clinics or hospitals) and through an online survey link (Qualtrics survey software), which was sent to their email addresses. The two procedures enhanced our ability to recruit physicians who are difficult to reach/invite due to time constraints or geographical distance. The questionnaire was administered to the physicians who agreed to participate after explaining the study’s objectives. A participants’ information sheet was provided for further clarification of the study. In addition, they were informed that completing the questionnaire was considered written consent to take part in the study.

### The questionnaire tool

Previously validated questionnaires developed by Workneh et al. (2016) and Khazzaka (2019) were used in this study to explore the characteristics of medical promotion and the influence of MRs on the prescribing practice of physicians [15, 19]. The use of a pre-existing questionnaire had the advantage of using a validated and tested instrument, which increased its reliability. In addition, it allowed comparison between different study populations. The questionnaire tool adopted from Khazzaka was previously pre-tested in the original study on 29 physicians from two hospitals in Lebanon to assess whether the questionnaire tool was filled out properly, understood, and whether the questions reflected the main points they were trying to cover and addressed their objectives. This pilot study confirmed that the questions were clear and easy to understand. However, details about the validity of the other questionnaire tool developed by Workneh et al. were not meniotned in the original study.

The questionnaire tool comprised nine sections: demographics and practice characteristics (5 items, multiple choice questions (MCQ) format), frequency of visits and length of discussions between drug promoter and physicians in the last 12 months (3 items, MCQ format), frequency and kinds of marketing promotional tools offered to physicians (3 items, one 5-point Likert scale ranging from never to always and two items in MCQ format), characteristics of drug information provided by MRs (2 items, one 5-point Likert scale ranging from never to always and one item in MCQ format), area of focus and attitude of MRs regarding competitors’ products (2 items, MCQ format), references used by physicians in case of problem during prescribing (1 item, MCQ format), pharmaceutical marketing strategies’ influence on physicians’ prescribing patterns (11 items, 5-point Likert scale ranging from never to always), prescribing practices (2 items, yes/no format), and gift acceptance and ethical norms (9 items, yes/no format).

The forward and backward translation technique was used for translation of the study questionnaire into the Arabic language. The forward translation focused on conceptual translation and was completed by two pharmacists independently whose first language was Arabic. The final Arabic version that was established through the forward translation was followed by a backward translation. Finally, the back-translated draft of the questionnaire was compared to the original questionnaire developed by Workneh et al. and Khazzaka. This was followed by a pilot study on 20 physicians in Jordan and Iraq, who were asked to complete the questionnaire tool face-to-face and were asked if any of the questions were considered unacceptable or offensive or difficult to understand; they confirmed that the questions were easy to understand and reflected the study objectives.

### Sample size

Based on the latest available statistics in Jordan and Iraq and assuming that the total number of registered physicians was approximately 30,000 in each country, using a confidence interval of 95%, standard deviation of 0.5, and margin of error of 5%, the minimum required sample size was 380 physicians from each country. A total of 801 physicians completed the questionnaire in this study. Of these, 401 were Jordanian and 400 were Iraqi physicians.

### Statistical analysis

Study data were analysed using statistical package for social sciences (SPSS) software, version 25. Continuous data were reported as mean (μ) ± SD. Categorical data were used to describe the participants’ basic demographic information and reported as frequencies and percentages. Chi-square test and Fisher’s test (less than 10 observations) were applied to compare the attitude of physicians in Jordan and Iraq and the level of significance was predetermined as 5%.

## Results

### Characteristics of study participants

A total of 850 physicians were approached during the study period, of which a total of 801 physicians completed the questionnaire (response rate 94.2%). Of these, 401 were Jordanian and 400 were Iraqi physicians (Table 1). The mean age of the study participants was 45.9 years (SD: 10.4). The majority of the respondents were male (68.0%). The highest proportion of the participants were specialist physicians (74.4%) with more than 10 years’ experience (66.7%) working in both public and private clinics (57.1%) and who used mainly medical textbooks (51.3%) followed by academic journals (44.9%) when experiencing problems when prescribing. For further details, refer to Table 1.

**Table 1:** Demographic and practice characteristics of study participants

Variable	Overall (*n*= 801)	Jordan (*n*= 401)	Iraq (*n*= 400)	***P***-value
**Age** (years) (mean (SD))	45.9 (10.4)	45.1 (9.2)	46.6 (11.5)	**0.037**
**Gender**				
Males	545 (68.0%)	274 (68.3%)	271 (67.8%)	0.460
**Years of experience**				
Less than 5 years	89 (11.1%)	30 (7.5%)	59 (14.8%)	**0.004**
6 – 10 years	178 (22.2%)	96 (23.9%)	82 (20.5%)	
More than 10 years	534 (66.7%)	275 (68.6%)	259 (64.8%)	
**Qualification**				
General practitioner	205 (25.6%)	69 (17.2%)	136 (34.0%)	**0.000**
Specialist	596 (74.4%)	332 (82.8%)	264 (66.0%)	
**Practice site**				
Public healthcare	144 (18.0%)	54 (13.5%)	90 (22.5%)	**0.003**
Private clinic	200 (25.0%)	111 (27.7%)	89 (22.2%)	
Both	457 (57.1%)	236 (58.8%)	221 (55.3%)	
***References used by healthcare professionals during their daily life practices for prescribing:*** (more than one answer can be chosen)				
Consultation of drug promoters	120 (15.0%)	31 (7.7%)	89 (22.3%)	**0.000**
Pharmaceutical company drug guides	277 (34.5%)	105 (26.1%)	172 (43.0%)	
Medical text books	411 (51.3%)	177 (44.1%)	234 (58.5%)	
Academic journals	360 (44.9%)	167 (41.6%)	193 (48.4%)	
Consultation with specialist doctor	230 (28.7%)	91 (22.7%)	139 (34.8%)	
Consultation with other GPs	129 (16.1%)	66 (16.4%)	63 (15.8%)	

### Frequency of visits and promotional methods

As shown in Table 2, most of the physicians reported that the frequency of MR visits to their clinics or healthcare centres was at least once weekly and up to 2–3 times per week (31.0 and 26.1%, respectively), spending “less than or equal to 10 minutes” and “11 to 20 minutes” during their drug promotion visits (47.2 and 46.2%, respectively). Face-to-face meetings (76.5%) followed by medical samples (52.6%) were the two promotional methods most commonly used by MRs during their visits. There was a statistically significant difference regarding the use of promotional methods between Jordan and Iraq. Face-to face visits were more commonly reported in Iraq (*p* < 0.001), while using brochures and stickers and referring to different articles were more common in Jordan (p < 0.001).

**Table 2:** Characteristics of pharmaceutical promotion

Variable	Overall	Jordan	Iraq	***P***-value
**How many times do MRs visit you for drug promotions?**				
Daily	117 (14.6%)	11 (2.7%)	106 (26.5%)	**0.000**
One time per week	248 (31.0%)	135 (33.7%)	113 (28.2%)	
2 – 3 times per week	209 (26.1%)	133 (33.2%)	76 (19.0%)	
2 times per month	135 (16.9%)	51 (12.7%)	84 (21.0%)	
Occasionally	85 (10.6%)	64 (16.0%)	21 (5.3%)	
Never^a^	7 (0.9%)	7 (1.7%)	0	
**What is the length of the discussion during their drug promotion (in minutes):** (*n*= 799)				
Less than or equal to 10 minutes	378 (47.2%)	172 (42.9%)	206 (51.5%)	**0.019**
11 – 20 minutes	370 (46.2%)	195 (48.6%)	175 (43.8%)	
More than or equal to 21 minutes	51 (6.4%)	32 (8.0%)	19 (4.8%)	
**What are the promotional methods used by MRs during their visits?** (more than one answer could be chosen)				
Face-to-face visits	613 (76.5%)	281 (70.1%)	332 (83.0%)	**0.000**
Using brochures and stickers	330 (41.1%)	192 (47.9%)	138 (34.6%)	**0.000**
Using medical samples	421 (52.6%)	202 (50.4%)	219 (54.8%)	0.229
Using electronic materials	165 (20.6%)	74 (18.5%)	91 (22.8%)	0.133
Referring to different articles	114 (14.1%)	76 (19.0%)	38 (9.6%)	**0.000**
Participating in new product launches	105 (13.1%)	44 (11.0%)	61 (15.3%)	0.073
Participating in company cycle meetings	75 (9.4%)	43 (10.7%)	32 (8.0%)	0.186

^a^Fisher test was applied

Pharmaceutical promotion was not consistent between Jordan and Iraq. Frequency of visits by MRs, length of MR visits and type of promotional methods used in Jordan and Iraq were not the same and differed significantly (*p* < 0.01).

### Kinds of marketing promotional tools

Table 3 shows that 48.1% of the participating physicians reported that they would accept the marketing promotional tools offered to them. The vast majority of the participating physicians (86.8%) reported that they received marketing promotional tools from MRs during their visits. The most commonly offered marketing promotional tools were drug samples (63.2%), coffee cups (31.7%), and invitations for lectures accompanied by dinner (28.0%). For further details, refer to Table 3.

**Table 3:** Characteristics of promotional tools used in pharmaceutical promotion

Variable	Overall	Jordan	Iraq	***P***-value
**Do you accept marketing promotional tools offered by MRs?**				
Yes	385 (48.1%)	185 (46.1%)	200 (50.0%)	0.274
**Frequency of marketing promotional tools offered by MRs:**				
Always	37 (4.6%)	14 (3.5%)	23 (5.8%)	**0.000**
Frequently	225 (28.1%)	77 (19.2%)	148 (37.0%)	
Occasionally	249 (31.1%)	133 (33.2%)	116 (29.0%)	
Rarely	184 (23.0%)	104 (25.9%)	80 (20.0%)	
Never	106 (13.2%)	73 (18.2%)	33 (8.3%)	
**Kind of marketing promotional tools offered by MRs:** (more than one answer can be chosen)				
Lectures accompanied by dinner invitation	224 (28.0%)	93 (23.2%)	131 (32.8%)	**0.003**
Drug samples	506 (63.2%)	238 (59.4%)	268 (67.0%)	**0.025**
Stationery	59 (7.4%)	32 (8.0%)	27 (6.8%)	0.505
Coffee cups	254 (31.7%)	137 (34.2%)	117 (29.3%)	0.135
Sponsored education	176 (22.0%)	87 (21.6%)	89 (22.3%)	0.839

There was no statistically significant difference in terms of physicians’ attitude toward accepting marketing promotional tools offered by MRs in Jordan and Iraq (*p* = 0.274). However, there was a statistically significant difference between Jordan and Iraq in terms of the frequency of offering marketing promotional tools and the specific types of promotional tools offered (*p* < 0.05).

### Type and accuracy of MR information

More than half of the participating physicians reported that MRs mainly focus on the price and the brand name of the product during their medical promotion. Nearly half (48.8%) of the participating physicians reported that MRs were frequently accurate in the information they presented during medical promotions. For further details, refer to Table S1.

When we asked the participating physicians about what type of information MRs focus on during their medical promotions, we found that in Jordan and Iraq there was no significant difference in terms of providing information about brand name of the product, drug interaction, and treatment precautions (*p* > 0.05), which highlights that these were important points discussed during medical promotions regardless of the differences between the two countries. However, in terms of other types of medical promotion, we found that there was a statistical difference between the two countries (*p* < 0.05). Additionally, the accuracy of providing this information by MRs differed significantly and was higher in Jordan (p < 0.05).

### Attitudes of medical representatives

According to most of the physicians who participated in the study, most MRs focused on the key selling points of their product (38.7%) during medical promotions. Additionally, 39.6% of the physicians reported that MRs had a negative attitude toward their competitors’ products, while 30.5% were neutral. For further details, refer to Table S2.

When we asked about the focus of MRs during their medical promotions in Jordan and Iraq, we found that their focus was not the same, and the key messages during their visits to physicians differed significantly between the two countries (*p* < 0.05). In Jordan, MRs were more oriented toward the selling points of their product and the formulation advantage, whereas in Iraq, the MRs were more oriented toward the differential advantage and the scientific background (*p* < 0.05).

Regarding the attitude of MRs toward competitors’ products, there was a significant difference between the two countries. In Jordan, nearly 50% of the physicians surveyed reported that all or most of the MRs have a negative attitude toward competitors’ products.

### Influence of marketing strategies on prescribing practices

MR visits, offering drug samples, sales calls made by pharmaceutical companies, promotional drug brochures, medical equipment (gifts), and travel sponsorship, conferences or sponsorship for personal tours are the most commonly reported marketing strategies that have an influence on physicians’ prescribing practices compared to other strategies.

**Fig. 1:**
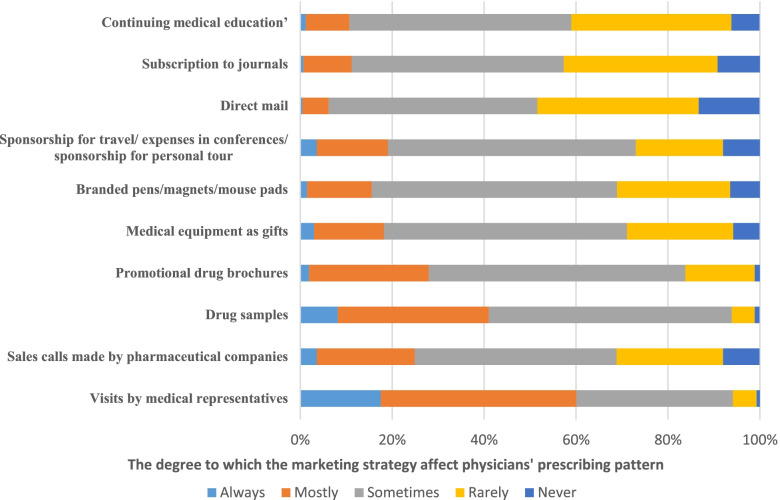
The influence of promotional tools on physicians prescribing practices

Among the different marketing strategies that pharmaceutical companies use to influence physicians’ prescribing practices, the most influencing tool, as reported by physicians, was visits by MRs. Fig. 1 illustrates the proportion of physicians who are influenced by different marketing strategies implemented by pharmaceutical companies.

### Acceptance of marketing strategies

Table S3 shows physicians’ attitudes toward accepting a shift in their prescribing practices from one company to another: 69.2% reported that they would follow this practice “if both drugs were generic”. Around 70% of the physicians reported that they would change their clinical practice after participating in conferences or attending meetings. The majority of physicians (77.6%) reported that they would accept low-cost gifts (pens, magnets, mouse pads) for drug promotion from pharmaceutical companies. However, 52.6% did not recommend the continuous supply of (low-cost) gifts to the physician regularly (at every visit) by the MR as they did not find it justifiable.

Around half of the physicians (47.9%) reported that they think that recreational gifts (high-cost, such as laptops, mobiles and LCDs) are justifiable in drug promotion. However, 49.7% of them could not justify the continuous supply of high-cost gifts to the physician on a regular basis (at every visit) by the MR. Around 81.9% of the physicians agreed that pharmaceutical companies could invite doctors to international congresses.

More than half of the physicians (54.2%) reported that they used free medical samples to treat their patients. The majority (92.0%) of the physicians reported that there was a need for a strengthening of ethical standards to control the interaction between physicians and the pharmaceutical industry, and that MRs should have credentials of professional and ethical capability to execute their profession (97.0%). Additionally, 96.3% of the physicians reported that they consider it right to regulate the number of visits by MRs to physicians. For further details, refer to Table S3.

Regarding the influence of medical promotion and other promotional activities of pharmaceutical companies on physicians’ practices, there was a significant difference between Jordan and Iraq in terms of shifting drug prescribing from one company to another (if both drugs are generic), acceptance of low- and high-cost gifts, and usage pattern of medical samples (*p* < 0.01). However, there was no statistically significant difference between physicians in Jordan and Iraq in terms of changing their clinical practice after attending meetings or conferences (*p* = 0.499).

Despite the fact that in both Jordan and Iraq the majority of participating physicians agreed on the need for a strengthening of ethical standards to control the interaction between physicians and pharmaceutical companies, the necessity of certifying MRs’ professional and ethical capabilities, and regulating number of MR visits, these attitudes were not the same in the two countries and differed significantly. In Jordan, they were more interested in strengthening of ethical standards, while in Iraq they were more interested in certifying MRs and regulating number of MR visits (*p* < 0.01).

## Discussion

Pharmaceutical companies in Jordan and Iraq, as in almost all countries, urge physicians to prescribe their pharmaceutical products due to the increasing number of pharmaceutical companies and the intense competition between them. The marketing efforts by MRs and other advertising tools are considered the main factor influencing physicians’ prescribing practices. Previous studies have found a direct correlation between pharmaceutical promotional tools and physicians’ prescribing practices [13, 16, 18, 20, 21].

Our study confirmed the findings of previous studies and found that physicians are influenced by pharmaceutical companies’ promotional techniques to prescribe promoted drugs. Over two-thirds (69.2%) of the participating physicians reported that MRs have an impact on physicians’ prescribing practices. In 2014, an Iraqi study reported that 77% of physicians preferred to prescribe newer medications presented to them by MRs [18]. A previous study in Yemen found that the majority of the physicians believed that they were under marketing pressure to prescribe certain medicines due to the promotional methods used by pharmaceutical companies [14]. Similar findings were reported in previous studies conducted in other countries such as Turkey, United Kingdom, and the United States [20, 22–24]. Physicians practice characteristics including working in primary healthcare centres (and having high work load) and having 5 years of experience or lower were important predictors that affected physicians prescribing practices significantly [20].

MRs receive one-third of total marketing expenses and are considered the key promotional tool of the pharmaceutical industry [12]. Previous literature has reported that physicians consider MRs as “information providers”; however, in this study, the participating physicians reported the use of medical textbooks and academic journals as references in case of problems during prescribing, and said that they considered MRs to be information providers for new drugs. A previous study that was conducted in Turkey reported that there is a lack of continuous medical education provided by the public sector to practicing physicians, and 53.0% of the participating physicians reported that they only participated in in training courses of pharmaceutical companies [20]. This increases physicians’ reliance on information provided by pharmaceutical companies solely. Commercial information makes up provided by pharmaceutical companies for the lack of training in healthcare services, and this is even more common in developing countries where the drug industry influence is greater [25, 26]. This source of information could lead to poor prescribing habits if implemented improperly [27, 28].

Our study shows that visits of MRs (face-to-face visits) (76.5%) are the main promotional tool that influence physicians’ prescribing practices due to the relationship built between physicians and MRs. Additionally, most of them reported their preference for receiving information directly from MRs and having discussions and interactions. They also used the new medication directly after they had compared it with other products and possessed evidence about it. This was clearly demonstrated when the frequency of visits by MRs was high and on monthly basis [20]. Our findings confirmed this and found that frequent MR visits certainly affect physicians’ prescribing practices. This could be due to the fact that face-to-face visits encourage reliability and trust [20]. Physicians are also concerned about patients’ safety and a face-to-face explanation may give them more reassurance about the new product. Nonetheless, some studies have shown that MRs have a minimal effect on physicians’ prescribing practices in the US and some European countries. They have reported that the effect of MRs on the prescribing practices of physicians might be minimal or absent [29]. These results may indicate different influences due to the differences in the healthcare systems and regulations that guide prescribing and promotion of medical products between different countries [12, 13, 18].

Regarding gift acceptance, it has been shown that most physicians (86.8%) have received marketing promotional tools from MRs during their visits; a large number of physicians (63.2%) claimed to have accepted gifts and marketing tools, mainly drug samples to use with their patients or as a reminder, which confirms the results of previous studies. A previous study in the United States reported that 61% of the participating physicians received meals, tickets to entertainment events, or free travel from pharmaceutical companies, and 13% received various types of benefit including financial incentive [30]. A recent study in Sudan showed that 91.2% of the participating physicians received drug samples and considered that receiving them was ethically acceptable and a benefit for their patients who cannot afford the medicines [12, 18, 19, 31].

Concerning gifts with a high cost, this study showed that just over half of Jordanian and Iraqi physicians consider their acceptance as unethical (52.1%). The same applies to the continuous supply of high-cost gifts (49.7%). In a previous study in Lebanon, 74.8% of the participating physicians considered the acceptance of expensive gifts inappropriate and unethical, and in Libya, only 10% of physicians were comfortable with accepting such gifts. It is very important to mention that gifts, whether they are cheap or expensive, cost the pharmaceutical company money that surely will be added to the price of the medication [18, 19, 31].

The vast majority (92.0%) of the physicians in our study mentioned that there is a need to strengthen ethical norms to regulate the physician-pharmaceutical industry interaction, and that MRs should have professional and ethical certification to practise their profession [32]. This confirms a study in India, where 80.2% of physicians thought the same [33]. This view should inform pharmaceutical companies that their relationships with physicians must be modified, because while many physicians admit that gifts and frequent MR visits are considered to be an expression of appreciation, they also influence the prescribing of specific products. This is important in Middle Eastern and low socioeconomic countries where physicians’ salaries are not good. Therefore, the influence of pharmaceutical companies and their gifts may be observed even more [34].

Continuing medical education conferences and free medical samples also produce a positive and significant impact on preference for particular pharmaceutical companies’ products. Such products may provide financial benefits to patients and may help patients who cannot pay for their drugs. Physicians are aware that receiving free medical samples impacts their prescribing practices. However, they believe that drug samples are ethical if they benefit the patients [12, 18]. Additionally, 96.3% of the physicians reported that the number of visits of MRs to physicians should be regulated to cause less inconvenience, not exhaust the physicians in terms of taking up a lot of their time, reduce the high number of weekly visits, and restrict discussions on topics not related to the matter in hand.

In our study, 69.9% of the physicians reported that they would change their clinical practice after participating in conferences or meetings, and 81.9% of them agreed that pharmaceutical companies can invite physicians to international congresses. In 2014, a study in Peru reported that 88% of respondents believed that receiving gifts and attending lunches sponsored by pharmaceutical firms did not affect their prescribing practices [35].

About 40% of the participating physicians in this study reported that MRs had a negative attitude toward competitors’ products, compared to 30.5% who were neutral. A negative attitude toward competitors’ products could be defined as ignoring the advantages and only paying attention to the weaknesses of competitive products. Although it is not clear whether the MRs’ attitude toward competitors affects prescribing practices, physicians confirmed that a positive reaction and mutual respect between competing MRs led to a positive view of the companies and their products.

Based on the findings of our study, it is recommended that laws should be structured to include ethical guidelines for drug promotion, enabling monitoring and encouraging compliance by pharmaceutical companies. This will ensure that medical promotion provided by pharmaceutical companies is solely aimed at providing physicians with the latest information on pharmaceutical products available in the market and their most suitable indications, without influencing their prescribing practices. This would lead to better health outcomes for the patients. Ethical training for MRs is always recommended, with more focus on ethical courses during pharmacy undergraduate studies. Additionally, it is important to have more implementation of medical and clinical guidelines, which makes the margin for choosing different brands of medications very narrow, therefore leading to more safety and better outcomes.

There are a limited number of studies that have addressed this topic globally, and very few of them have been in Middle Eastern countries. This study is among the first in the Middle East region to explore the acceptability of medical promotion tools and their influence on physicians’ prescribing practices. This study examined the characteristics of medical promotion provided by pharmaceutical companies through their MRs to physicians in Jordan and Iraq. It provides detailed information about the characteristics of medical promotion in Jordan and Iraq and its influence on physicians’ prescribing practices. We did not restrict our sample to physicians working in specific healthcare settings or specific specialities. However, this study does have some limitations. Military physicians were not included in this study due to restricted access. In addition, the use of convenience sampling might have affected the generalisability of our findings as a non-probability sampling technique was employed in this study. Some of the promotional concepts used were unknown to some physicians and the questionnaire was anonymous, which may lead to desirability bias. To minimise the possibility of this common bias we distributed the study questionnaire extensively to cover the majority of the working physicians from different specialities in different healthcare settings in Amman and Baghdad (the capital cities of Jordan and Iraq, where the majority of registered physicians practise). This should increase the generalisability of our findings. Despite the fact that in the original study Workneh et al. reported that they used a structured, pre-tested instrument, there was no information on the psychometric properties and the validity of the instrument; therefore, our findings should be interpreted carefully.

## Conclusion

Medical promotion tools have a clear influence on physicians’ prescribing practices in Jordan and Iraq. Therefore, medical promotion should be controlled and guided by clear and country-specific ethical guidelines. This will ensure safe medical promotion to physicians and optimise healthcare practices provided to patients.

## Supplementary Information

**Additional file 1.**

## Data Availability

The datasets used and/or analysed during the current study are available from the corresponding author on reasonable request.

## Abbreviations

MR: Medical Representative
SPSS: Statistical Package for Social Sciences
SD: Standard Deviation
MCQ: Multiple Choices Question
