# Supplementary material for: The attitude and acceptability towards medical promotional tools and their influence on physicians’ prescribing practices in Jordan and Iraq: a cross-sectional study
Source: BMC Health Serv Res. 2022 Jan 25;22:105. doi: 10.1186/s12913-022-07525-1 (PMC8790844; doi:10.1186/s12913-022-07525-1)
Supplement: Supplementary file 1 — Additional file 1. [file 12913_2022_7525_MOESM1_ESM.docx]

**Supplementary material:**

- Table S1. Characteristics of information provided by medical representatives.

- Table S2. Medical representative visits focus and attitudes towards competitors.

- Table S3. Gift acceptance among physicians and their perception towards and recommendations for pharmaceutical promotion.

**Table S1.** Characteristics of information provided by medical representatives

| **Variable** | **Overall** | **Jordan** | **Iraq** | **P-value** |
| --- | --- | --- | --- | --- |
| **What type of information do MRs focus on during their medical promotion?**  (more than one answer can be chosen) | | | | |
| Approved drug indication | 289 (36.1%) | 175 (43.6%) | 114 (28.5%) | **0.000** |
| Brand name of the product | 430 (53.7%) | 212 (52.8%) | 218 (54.5%) | 0.537 |
| Drug interaction | 274 (34.2%) | 134 (33.4%) | 140 (35.1%) | 0.553 |
| Side effects | 178 (22.2%) | 77 (19.2%) | 101 (25.3%) | **0.040** |
| Price of the product | 459 (57.3%) | 188 (46.9%) | 271 (67.8%) | **0.000** |
| Contraindications | 281 (35.1%) | 110 (27.4%) | 171 (42.8%) | **0.000** |
| Precautions | 50 (6.2%) | 21 (5.2%) | 29 (7.2%) | 0.239 |
| **To what extent is the information given by MRs adequate and accurate?** | | | | |
| Always | 27 (3.4%) | 16 (4.0%) | 11 (2.8%) | **0.002** |
| Frequently | 391 (48.8%) | 220 (54.9%) | 171 (42.8%) |  |
| Occasionally | 211 (26.3%) | 88 (21.9%) | 123 (30.8%) |  |
| Rarely | 167 (20.8%) | 73 (18.2%) | 94 (23.5%) |  |
| Never§ | 5 (0.6%) | 4 (1.0%) | 1 (0.3%) |  |

§ Fisher test was applied

**Table S2.** Medical representative visits focus and attitudes towards competitors

| **Variable** | **Overall** | **Jordan** | **Iraq** | **P-value** |
| --- | --- | --- | --- | --- |
| **What is the focus of MRs during their medical promotions?** | | | | |
| Selling point of their product | 310 (38.7%) | 172 (42.8%) | 138 (34.5%) | **0.003** |
| Differential advantage | 242 (30.2%) | 110 (27.4%) | 132 (33.0%) |  |
| Scientific background | 66 (8.2%) | 21 (5.2%) | 45 (11.3%) |  |
| Formulation advantage | 183 (22.8) | 98 (24.4%) | 85 (21.3%) |  |
| **What is the attitude of MRs towards competitors’ products?** | | | | |
| They all had a negative attitude | 87 (10.9%) | 35 (8.7%) | 52 (13.0%) | **0.018** |
| Most of them had a negative attitude | 317 (39.6%) | 181 (45.1%) | 136 (34.0%) |  |
| Most of them were neutral | 244 (30.5%) | 111 (27.7%) | 133 (33.2%) |  |
| Almost none of them had a negative attitude | 153 (19.1%) | 74 (18.5%) | 79 (19.8%) |  |

**Table S3.** Gift acceptance among physicians and their perception towards and recommendations for pharmaceutical promotion

| **Variable** | **Overall** | **Jordan** | **Iraq** | **P-value** |
| --- | --- | --- | --- | --- |
| ***Prescribing pattern*** | | | | |
| **Shifting drug prescribing from one company to another (if both drugs are generic)** | | | | |
| Yes, always | 154 (19.2%) | 82 (20.4%) | 72 (18.0%) | **0.000** |
| Yes, sometimes | 400 (49.9%) | 151 (37.7%) | 249 (62.3%) |  |
| No | 247 (30.8%) | 168 (41.9%) | 79 (19.8%) |  |
| **Changing clinical practice after attending meetings or conferences** | | | | |
| Yes, always | 164 (20.5%) | 76 (19.0%) | 88 (22.0%) | 0.499 |
| Yes, sometimes | 396 (49.4%) | 199 (49.6%) | 197 (49.3%) |  |
| No | 241 (30.1%) | 126 (31.4%) | 115 (28.7%) |  |
| ***Gift acceptance*** | | | | |
| **Acceptance of low-cost gifts for drug promotion** | | | | |
| Yes, always | 126 (15.7%) | 57 (14.2%) | 69 (17.3%) | **0.000** |
| Yes, sometimes | 496 (61.9%) | 219 (54.6%) | 277 (69.3%) |  |
| No | 179 (22.3%) | 125 (31.2%) | 54 (13.5%) |  |
| **Do you find it justifiable to receive low-cost gifts at every visit of the medical representative?** | | | | |
| Yes, always | 79 (9.9%) | 43 (10.7%) | 36 (9.0%) | **0.000** |
| Yes, sometimes | 301 (37.6%) | 121 (30.2%) | 180 (45.0%) |  |
| No | 421 (52.6%) | 237 (59.1%) | 184 (46.0%) |  |
| **Acceptance of high-cost recreational gifts for drug promotion** | | | | |
| Yes, always | 57 (7.1%) | 12 (3.0%) | 45 (11.2%) | **0.000** |
| Yes, sometimes | 327 (40.8%) | 156 (38.9%) | 171 (42.8%) |  |
| No | 417 (52.1%) | 233 (58.1%) | 184 (46.0%) |  |
| **Do you find it justifiable to receive high-cost gifts at every visit of the medical representative?** | | | | |
| Yes, always | 64 (8.0%) | 14 (3.5%) | 50 (12.5%) | **0.000** |
| Yes, sometimes | 339 (42.3%) | 143 (35.7%) | 196 (49.0%) |  |
| No | 398 (49.7%) | 244 (60.8%) | 154 (38.5%) |  |
| **The usage pattern of free medical samples** (more than one answer can be chosen) | | | | |
| As a reminder | 308 (38.5%) | 134 (33.4%) | 174 (43.5%) | **0.002** |
| To treat people | 434 (54.2%) | 242 (60.3%) | 192 (48.0%) |  |
| For personal use | 59 (7.4%) | 25 (6.2%) | 34 (8.5%) |  |
| **Is there a need for a strengthening of ethical standards to control the interaction between physicians and pharmaceutical companies?** | | | | |
| Yes, always | 352 (43.9%) | 203 (50.6%) | 149 (37.3%) | **0.001** |
| Yes, sometimes | 385 (48.1%) | 169 (42.1%) | 216 (54.0%) |  |
| No | 64 (8.0%) | 29 (7.2%) | 35 (8.8%) |  |
| **Do you think that MRs should have a certificate of professional and ethical capability to execute their profession?** | | | | |
| Yes, always | 487 (60.8%) | 211 (52.6%) | 276 (69.0%) | **0.000** |
| Yes, sometimes | 290 (36.2%) | 172 (42.9%) | 118 (29.5%) |  |
| No§ | 24 (3.0%) | 18 (4.5%) | 6 (1.5%) |  |
| **Would you consider it right to regulate the number of visits of MRs to doctors?** | | | | |
| Yes, always | 433 (54.1%) | 191 (47.6%) | 242 (60.5%) | **0.000** |
| Yes, sometimes | 338 (42.2%) | 186 (46.4%) | 152 (38.0%) |  |
| No§ | 30 (3.7%) | 24 (6.0%) | 6 (1.5%) |  |
| **Do you agree with pharmaceutical companies inviting doctors to international congresses?** | | | | |
| Yes, always | 255 (31.8%) | 115 (28.7%) | 140 (35.0%) | **0.016** |
| Yes, sometimes | 401 (50.1%) | 199 (49.6%) | 202 (50.5%) |  |
| No | 145 (18.1%) | 87 (21.7%) | 58 (14.5%) |  |

§ Fisher test was applied
